# Assessment of physical activity level and quality of life in patients with chronic kidney disease in stages 3b and 4

**DOI:** 10.1590/1414-431X2026e15406

**Published:** 2026-11-09

**Authors:** F.T. dos Santos, C.G.S. Santos, F.C. Barreto, R. Fernandez

**Affiliations:** 1 Departamento de Fisiologia, Setor de Ciências Biológicas, Universidade Federal do Paraná, Curitiba, PR, Brasil; 2 Departamento de Clínica Médica, Setor de Ciências da Saúde, Universidade Federal do Paraná, Curitiba, PR, Brasil; 3 Serviço de Nefrologia, Complexo Hospital de Clínicas, Universidade Federal do Paraná, Curitiba, PR, Brasil

**Keywords:** Chronic kidney disease, Physical activity, Quality of life, CKD progression

## Abstract

The progressive decline in renal function in chronic kidney disease (CKD) is associated with multiple complications that affect the quality of life (QoL) of patients. However, physical activity may improve clinical outcomes. This study aimed to evaluate and correlate the physical activity levels of patients with CKD stages 3b and 4 with their QoL and risk of disease progression. This cross-sectional observational study included 50 patients with CKD stages 3b or 4. Physical activity levels were assessed using the International Physical Activity Questionnaire. QoL was evaluated using the Medical Outcomes Study 36-Item Short Form Health Survey (SF-36) questionnaire, combined with the sit-to-stand and handgrip strength tests. The risk of CKD progression was estimated based on the degree of albuminuria. Only 28% of the participants were classified as physically active. A significant association was observed between physical activity level and CKD stage. Most physically active participants had albuminuria of ≤30 mg/g, indicating a lower risk of CKD progression. Participants with A2 albuminuria were more likely to be inactive than those with A1 albuminuria (P=0.01). Additionally, active individuals demonstrated higher mean scores across most SF-36 domains and superior performance in the handgrip and sit-to-stand tests. A positive correlation was identified between physical activity and QoL, with the strongest correlations observed in the vitality (r=0.42; P<0.01) and general health (r=0.43; P<0.01) domains. Higher physical activity levels are associated with better QoL and lower albuminuria, suggesting a reduced risk of disease progression. These findings highlight the importance of promoting physical activity among patients with CKD stages 3b or 4.

## Introduction

Chronic kidney disease (CKD) is characterized by structural or functional abnormalities of the kidneys, particularly affecting their excretory function (1). In addition to excretion, other renal functions, such as hormone production, regulation of fluid and electrolyte balance, acid-base homeostasis, and blood pressure control, are compromised (1). Consequently, CKD is associated with a wide range of cardiovascular, endocrine, and musculoskeletal complications, leading to a significant decline in quality of life (QoL) and an increased risk of premature mortality (2). The condition is diagnosed when the glomerular filtration rate (GFR) falls below 60 mL/min/1.73 m^2^ for at least 3 months or when structural kidney damage is evident, regardless of GFR levels. Furthermore, CKD is classified based on cause, GFR category (G1-G5), and albuminuria category (A1-A3). A GFR value below 60 mL/min/1.73 m^2^ corresponds to stage G3a. Markers of kidney damage include albuminuria (>30 mg in a 24-h urine or albumin-to-creatinine ratio of >30 mg/g in a spot urine sample), imaging abnormalities, hematuria or leukocyturia, persistent electrolyte imbalances, and histopathological changes observed in renal biopsy (1,2).

In recent decades, the global prevalence of CKD has risen sharply, leading to growing concern within the international medical community (3). Between 1990 and 2017, CKD prevalence increased by 29.3%, with approximately 700 million individuals (9.1% of the global population) affected in 2017 alone. In 2017, CKD accounted for 1.28 million deaths, ranking it as the 12th leading cause of mortality worldwide (3). In Brazil, over 10 million individuals have CKD, with approximately 157,000 undergoing dialysis, an increase of more than 60% over the past decade (4).

Among modifiable risk factors, sedentary behavior and poor dietary habits play a prominent role (2). Lifestyle-related conditions, such as diabetes, which is responsible for 30-50% of CKD cases, highlight the relevance of behavioral interventions in disease prevention and management (1). In this context, regular physical activity has emerged as an effective non-pharmacological strategy. Regular physical activity improves blood pressure and glycemic control, mitigates disease-related symptoms, and enhances QoL, thereby slowing disease progression and improving prognosis (5).

Numerous studies have reported a positive association between physical activity level (PAL) and QoL in patients with CKD. Regular exercise contributes to better functional capacity (6), increased muscle strength (7), and reduction in symptoms, such as fatigue and depression (8), significantly enhancing patients' perceived well-being (9). Additionally, PAL may have a protective effect on CKD progression, possibly through modulation of inflammatory and oxidative stress pathways (10). Despite these benefits, physical inactivity remains prevalent in this population, particularly in those with CKD stages 3b and 4 (moderately to severely decreased GFR [30-44 mL/min/1.73 m^2^] and severely decreased GFR [15-29 mL/min/1.73 m^2^], respectively), who experience more severe complications and marked QoL impairment (11,12).

Pre-dialysis stages (stages 3b and 4) represent a critical period preceding the need for renal replacement therapy. In this context, further investigation of the relationship between PAL and QoL in this patient population is warranted. Such insights may help inform the development of targeted interventions aimed at promoting physical activity and improving clinical outcomes in this vulnerable group.

This cross-sectional study evaluated the impact of PALs on QoL and risk of CKD progression in patients with stages 3b and 4 undergoing outpatient care at a Brazilian university hospital. Our findings may support the development of personalized exercise interventions and evidence-based guidelines to encourage physical activity, ultimately contributing to improved QoL and prognosis in patients with advanced CKD.

## Material and Methods

### Study design

This was an observational, cross-sectional, quantitative study with a descriptive and correlational approach. Participants were recruited by convenience sampling from patients receiving care at the Chronic Kidney Disease outpatient clinic of the Hospital de Clínicas, Federal University of Paraná (UFPR), between June 2023 and January 2024. The study adhered to Resolution No. 466/12 of the National Health Council and was approved by the Research Ethics Committee of the Hospital de Clínicas - UFPR (Approval No. 5.946.537). All participants were informed about the study procedures and voluntarily signed an informed consent form. Participants were recruited as a convenience sample, including as many patients as possible given the routine flow of outpatient clinic visits.

### Inclusion and exclusion criteria

Inclusion criteria were patients diagnosed with CKD for at least 6 months, with GFR between 15 and 44 mL/min/1.73 m^2^ (stages 3b or 4), confirmed for at least 3 months and calculated using the CKD-EPI equation; age ≥18 years; and ability to provide informed consent.

Exclusion criteria were cognitive impairment preventing understanding of the questionnaires; inability to engage in regular physical activity or walk independently; and failure to attend the follow-up appointment.

A total of 60 patients initially completed the questionnaires; however, 10 were excluded because they missed the follow-up appointment, which included the physical tests.

### Clinical and laboratory parameters

The following parameters were assessed: PAL, QoL dimensions, and CKD progression risk. Social (e.g., sex, age, occupation, education) and clinical characteristics (e.g., CKD stage, history of hypertension, diabetes, smoking status, and body mass index - BMI) were retrieved from the patients' electronic medical records.

PAL was measured through interviews using the long version of the International Physical Activity Questionnaire (IPAQ), validated for older Brazilian adults by Benedetti et al. (13). This instrument evaluates frequency and duration of physical activity across various domains (household, leisure, occupational, commuting, and sitting time) over a typical week. Data were converted to minutes per week, and participants were classified as physically active if they met the recommendations of the Brazilian Physical Activity Guidelines: ≥150 min of moderate-intensity activity per week or ≥75 min of vigorous-intensity activity or an equivalent combination.

QoL was evaluated using the Medical Outcomes Study 36-Item Short Form Health Survey (SF-36), validated and translated into Portuguese (14). The SF-36 includes 36 items covering eight dimensions: functional capacity, physical role, emotional role, mental health, pain, vitality, social functioning, and general health. Each domain generates a score from 0 (worst) to 100 (best).

In addition to the SF-36, the “Functional Capacity” dimension was assessed objectively via two physical performance tests.

#### 30-Second sit-to-stand test

Assesses lower limb muscular endurance. Participants sat in a 45-cm-high chair with their back straight and feet flat on the floor, shoulder-width apart. They were instructed to rise to a full stand and return to sitting as many times as possible in 30 s, without using their arms. The total number of complete repetitions was recorded (15).

#### Handgrip strength test

Assesses upper limb strength using a SAEHAN SH5001 hydraulic dynamometer. Participants stood with arms extended and forearms in neutral rotation. Each participant completed three attempts per hand, with ∼1 min rest between trials. The best result from each hand was recorded (16).

CKD staging was determined using the CKD-EPI equation to calculate estimated GFR. A previous study involving CKD stage 3-4 patients undergoing aerobic training found similar eGFR values using creatinine or cystatin C, both at baseline and after an exercise protocol (17).

CKD progression risk was assessed via albuminuria levels, a recognized independent predictor of disease progression (2). Albuminuria was categorized as follows (albumin-to-creatinine ratio): A1: <30 mg/g (low risk); A2: 30-300 mg/g (moderate risk); A3: >300 mg/g (high risk).

### Data collection procedures

Data collection was conducted by a single, previously trained interviewer and divided into two stages: 1) Initial visit: administration of the IPAQ and SF-36 questionnaires through interview; and 2) Follow-up appointment: explanation and performance of the 30-second sit-to-stand test, followed by the handgrip strength test.

### Statistical analysis

Results are reported as means±SE. Descriptive statistics (frequency distributions) were used to characterize the sample. Chi-squared tests compared sociodemographic and clinical characteristics between active and inactive participants. The normality of SF-36 scores and physical test results was assessed using the Kolmogorov-Smirnov test. Depending on the data distribution, comparisons between active and inactive groups were performed using either the Student's *t*-test (parametric) or the Mann-Whitney U test (non-parametric). Associations between PAL and QoL scores were assessed using Spearman's correlation (for non-normally distributed variables) or Pearson's correlation (for normally distributed variables). A significance level of P<0.05 was adopted. Analyses were performed using GraphPad Prism 8.

## Results

The study included 50 patients with a mean age of 68.56±1.56 years. The mean PAL among the participants was 116±11.39 min of moderate and/or vigorous physical activity per typical week.

Table 1 presents the sociodemographic characteristics and PALs of the participants. Only 28% of the sample was classified as physically active. Most of the participants were males (54%), white (80%), married (58%), retired (80%), and had completed education up to high school level (54%). Although physical inactivity was proportionally more common among males, mixed-race individuals, and married participants, there were no statistically significant differences between the active and inactive groups in terms of sex, race, or marital status. However, statistically significant differences were found in relation to age group, occupation, and educational level. The mean age of physically active participants was significantly lower than that of the inactive ones (60.86±3.22 years *vs* 71.56±1.54 years, P=0.001).

**Table 1:** Assessment of IPAQ scores in relation to sociodemographic factors.

	n	Physically inactive		Physically active		P-value
		n	%	n	%	
Sample	50	36	72	14	28	-
Age group (years)						
40-49	3	1	33	2	67	**0.030**
50-59	9	4	44	5	56	
60-79	14	9	64	5	36	
70-79	16	15	94	1	6	
≥80	8	7	88	1	13	
Sex						
Male	27	20	74	7	26	0.723
Female	23	16	70	7	30	
Race						
White	40	28	70	12	30	0.529
Mixed race	10	8	80	2	20	
Marital status						
Married	29	23	79	6	21	0.176
Single	21	13	62	8	38	
Occupation						
Working	10	3	30	7	70	**0.001**
Not working	40	33	83	7	17	
Educational level						
Primary school	15	12	80	3	20	**0.005**
High school	27	22	81	5	19	
Higher education	8	2	25	6	75	

IPAQ: Physical Activity Questionnaire. P-values in bold indicate statistical significance; chi-squared test.

Table 2 summarizes the clinical characteristics of the participants according to their PALs. A statistically significant difference was found between the active and inactive groups regarding CKD stage (P=0.01), indicating a decrease in PAL as the disease progresses.

**Table 2:** Analysis of physical activity level in relation to clinical variables.

Variables	Physically inactive		Physically active		P-value
	n	%	n	%	
Sample	36	72	14	28	-
CKD stages					
3b	16	57	12	43	**0.010**
4	20	91	2	9	
Arterial hypertension	23	77	7	23	0.368
Diabetes mellitus	13	76	4	24	0.613
Smoking	15	83	3	17	0.181
BMI					
Underweight	0	0	1	100	0.404
Normal	13	81	3	19	
Overweight	9	60	6	40	
Obesity grade I	8	73	3	27	
Obesity grade II	5	83	1	17	
Obesity grade III	1	100	0	0	
Albuminuria levels					
A1	7	47	8	53	
A2	18	75	6	25	**0.010**
A3	11	100	0	0	

CKD: chronic kidney disease; BMI: body mass index. P-values in bold indicate statistical significance; chi-squared test.

Regarding comorbidities, the prevalence of diabetes mellitus and arterial hypertension was 34 and 60%, respectively. Eighteen participants reported being smokers. Although most hypertensive, diabetic, and smoking participants were inactive, no statistically significant differences were observed between the active and inactive groups regarding variables such as hypertension, diabetes mellitus, and smoking. Regarding BMI, overweight and obesity were prevalent, and participants with grades II and III obesity were more frequently inactive; however, this difference was not statistically significant (Table 2).

Most active individuals had albuminuria levels below 30 mg/g (A1), indicating a lower risk of CKD progression. Participants with albuminuria between 30 and 300 mg/g (A2) were more likely to be inactive than those with A1 albuminuria, and this difference was statistically significant (P=0.01) (Table 2).

Table 3 compares the active and inactive groups in terms of the SF-36 QoL dimensions and functional capacity test scores. The mean scores were higher in the active group across all the SF-36 domains, except for “emotional aspects”. Statistically significant differences were found in the domains of “functional capacity”, “physical role limitations”, “pain”, “general health”, “vitality”, “social functioning”, and “mental health”, indicating a better perceived QoL among physically active participants. Additionally, the active group had higher mean scores in the handgrip (Figure 1) and 30-second sit-to-stand (Figure 2) tests than the inactive group. These objective findings suggest that the active patients had significantly better functional capacity than the inactive patients.

**Figure 1:**
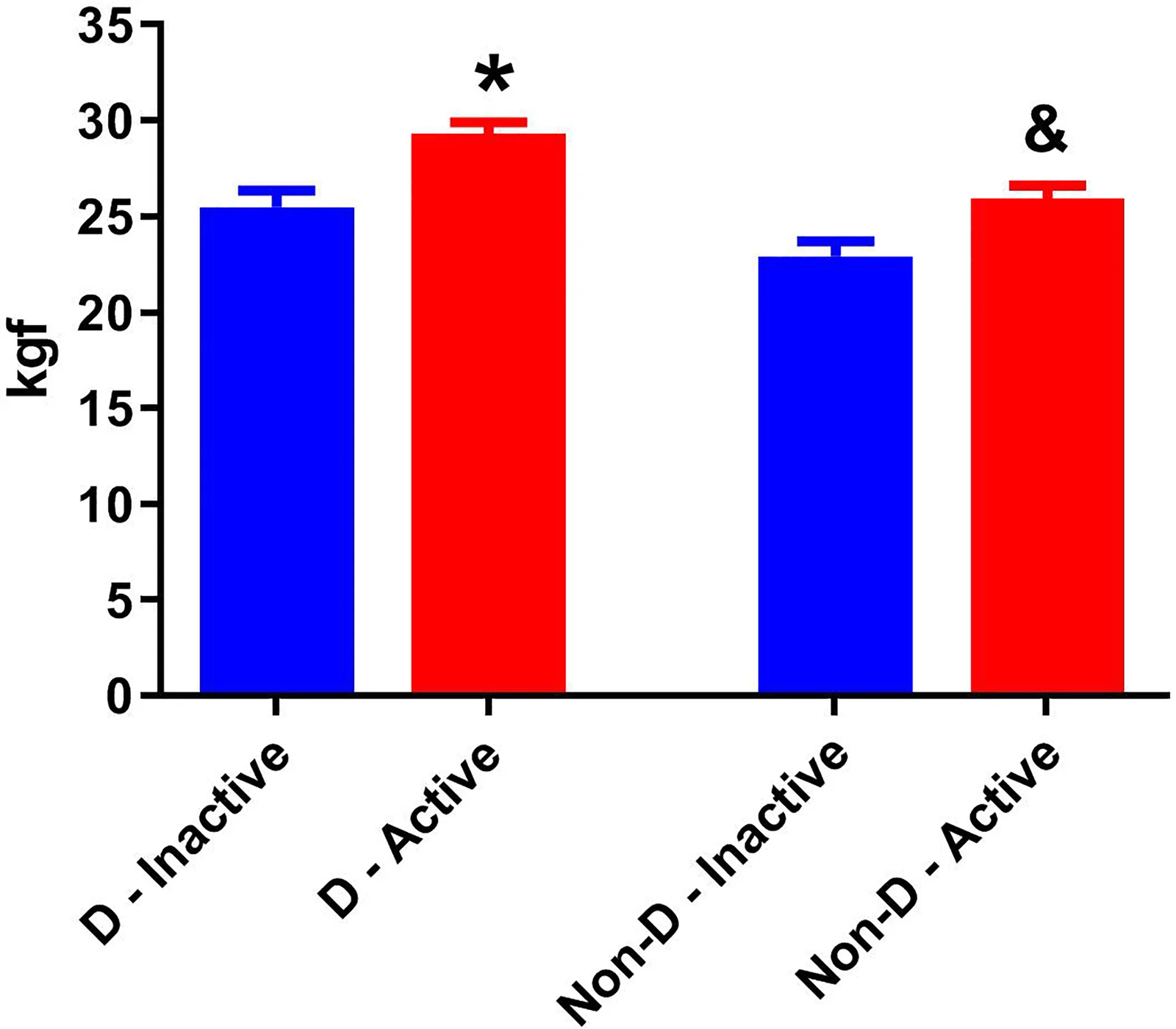
Results of the handgrip test in active and inactive patients. D: Dominant hand, Non-D: Non-dominant hand. Data are reported as means and SE. *P=0.001, ^&^P=0.006; Student's *t*-test.

**Figure 2:**
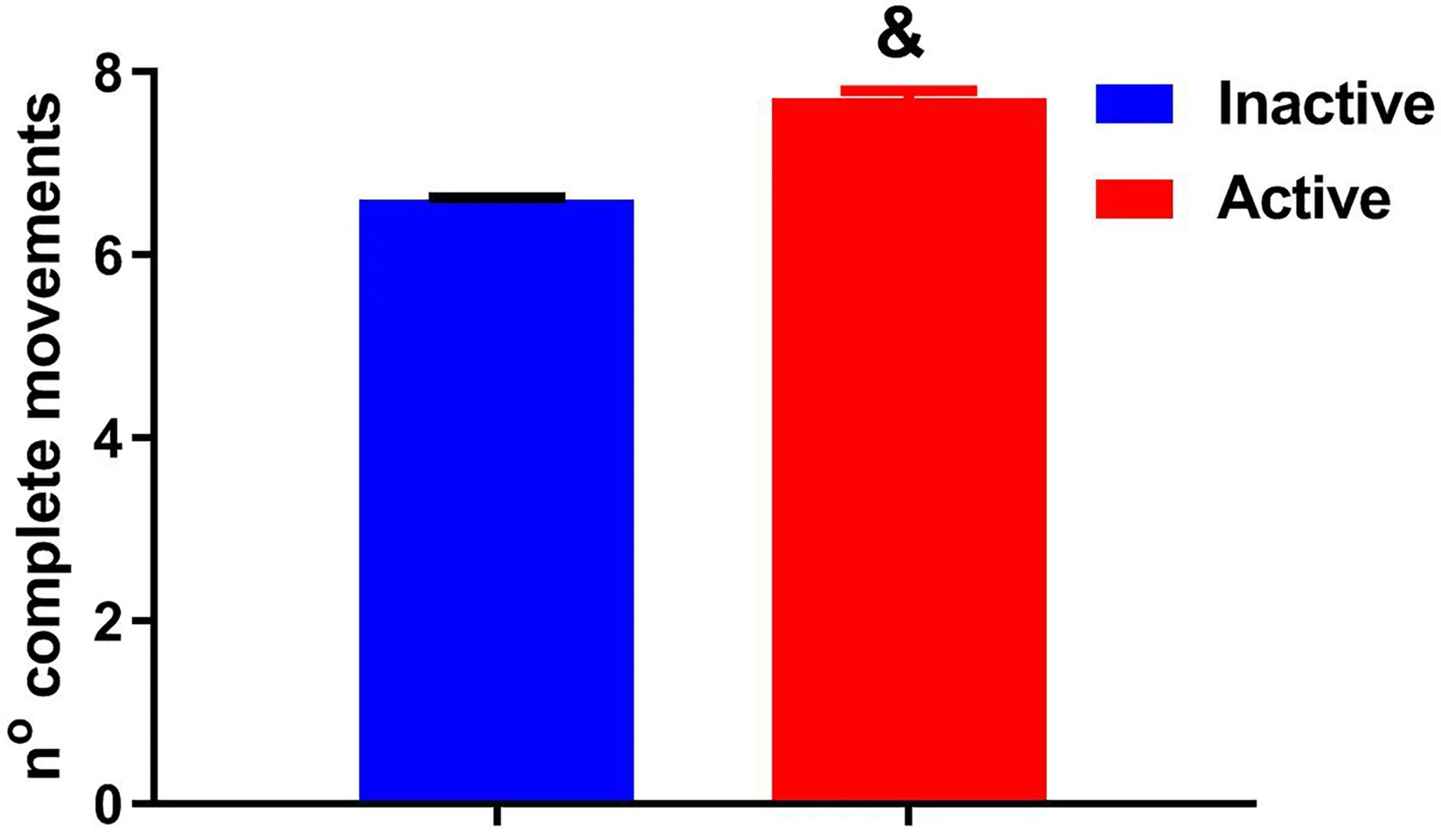
Results of the 30-second sit-to-stand test. Data are reported as means and SE. ^&^P=0.00374; Student's *t*-test.

**Table 3:** Comparison of the 36-Item Short Form Health Survey questionnaire (SF-36) dimensions and functional tests between active and inactive patients with correlation between each outcome and physical activity level determined using IPAQ.

		Active patients	Inactive patients	P-value	Correlation r	P-value
SF-36 questionnaire	Functional capacity	80.00±3.71	67.78±2.13	0.003*	0.3962	**0.004** ^#^
	Physical role	73.21±6.12	58.33±3.30	0.042*	0.22094	0.123^#^
	Pain	78.00±6.16	59.11±2.50	0.006*	0.31441	**0.026** ^#^
	General health	76.43±3.53	65.42±2.35	0.007*	0.4264	**0.002** ^#^
	Vitality	80.00±3.74	69.86±1.73	0.005*	0.42182	**0.001** ^#^
	Social functioning	75.00±4.14	74.31±4.51	0.037*	0.01591	0.912^#^
	Emotional role	81.01±9.69	88.13±2.66	0.596*	-0.08375	0.563^#^
	Mental health	80.57±5.97	58.67±4.15	0.002*	0.30421	**0.031** ^##^
Handgrip strength test (kgf)	Dominant hand	29.29±0.61	25.47±0.87	0.001**	0.38398	**0.005** ^##^
	Non-dominant hand	25.93±0.69	22.92±0.77	0.006**	0.32822	**0.019** ^##^
Sit-to-stand test		7.71±0.32	6.61±0.13	0.003*	0.41281	**0.002** ^##^

IPAQ: International Physical Activity Questionnaire. Data are reported as means±SE. P-values in bold indicate statistical significance; *Mann-Whitney U test (non-parametric); **Student's *t*-test; ^#^Spearman's correlation; ^##^Pearson's correlation.

Positive correlations were found between the PAL and five QoL domains (functional capacity, pain, general health, vitality, and mental health), with the strongest correlations in vitality (r=0.42; P<0.01) and general health (r=0.43; P<0.01) (Table 3). These results suggest that achieving adequate levels of physical activity may contribute to better perceived QoL in patients with CKD stages 3b and 4. Significant positive correlations were also observed between PAL and the objective functional capacity test results.

## Discussion

The findings of this study underscore important associations between PAL, QoL, and the risk of progression in patients with CKD stages 3b and 4. Physically active individuals demonstrated better QoL scores and a potentially reduced risk of CKD progression, as inferred from the lower levels of albuminuria.

The mean age of the participants was 68.6 years, which is in alignment with established epidemiological data that indicated a higher CKD prevalence in older adult populations (3,18). The predominance of males, whites, married, and retired individuals in the sample highlights the need for targeted intervention strategies tailored toward demographic subgroups to encourage physical activity and improve QoL.

Only 28% of the participants were classified as physically active, corroborating previous studies that reported physical inactivity as a common issue in CKD populations, including those in pre-dialysis stages (11,18,19). A statistically significant decline in PAL was observed with advancing CKD stage, reinforcing the importance of early interventions to mitigate health deterioration and prevent disease progression. Similar trends were observed in previous studies among patients in the UK, wherein physical activity decreased from 34% in stages 1 and 2 to only 6% in patients undergoing hemodialysis (19).

One contributing factor may be chronic exertional fatigue, defined as persistent debilitating exhaustion that limits daily activities, which affects up to 97% of individuals with CKD and emerges early in disease progression (20). This symptom may explain the low activity levels observed in 72% of the current study participants. In this regard, it should be mentioned that age and CKD intersect as risk factors for sarcopenia, frailty, and consequently, lower physical activity. The absence of a control group precluded us from better examining the potential individual effects of each condition. Despite this, considering the growing prevalence of CKD among older adults and the additive effects of both CKD and aging on frailty, our findings reinforce the importance of adopting an individualized approach for this population.

The association between higher CKD stages and lower physical activity may also, at least partially, be explained by the additive effects of both CKD and age on sarcopenia and frailty, which, in turn, lead to physical inactivity. CKD may contribute to the development of frailty or sarcopenia due to multiple factors such as inflammation, uremic toxins, and dietary restriction (20). In contrast, observational studies have suggested that worsening frailty is associated with an increased risk of CKD, whereas its amelioration is associated with a lower risk of CKD (21). Age is a key factor for CKD and frailty, and it is a non-modifiable risk factor (22). However, our study did not identify whether CKD or physical inactivity occurred first, nor did it evaluate the individual contributions of age and physical inactivity to CKD.

Regarding comorbidities, 34 and 60% of the participants had diabetes mellitus and hypertension, respectively, which are conditions widely associated with CKD (1,2). Although these conditions were more prevalent in the inactive group, the differences were not statistically significant. This suggests the need for multifactorial approaches to CKD management that include physical activity alongside medical treatment. Notably, a high prevalence of overweight and obesity was observed, as reflected in the BMI. This is consistent with prior evidence linking obesity to CKD incidence and progression (23). Lifestyle interventions, including physical activity and dietary counseling, are therefore critical, especially in early CKD stages (24). However, the BMI alone may underestimate obesity prevalence in patients with CKD; fat percentage measurements offer greater diagnostic accuracy, particularly in patients undergoing dialysis (23).

A significant association was identified between PAL and albuminuria, suggesting a potential protective effect of physical activity on renal function. Nevertheless, owing to the cross-sectional design of this study, causality cannot be established. Lower albuminuria may instead reflect better disease management, blood pressure control, and treatment adherence, all of which may enhance physical activity capacity and QoL. These findings align with previous studies that reported benefits of physical activity in both pre-dialysis and dialysis CKD populations (24-27).

A recent systematic review of 21 studies (published between 2007 and 2018) involving patients with pre-dialysis CKD undergoing exercise interventions reported improvements in aerobic and functional capacities, as well as enhanced QoL, even with low-to-moderate-intensity physical activity (26). However, the effects of physical activity on renal function remain inconclusive. Some studies have reported improved GFR and reduced proteinuria following aerobic or resistance training in stages 2-4 CKD (28-30), whereas others have shown benefits limited to reductions in inflammation and oxidative stress without significant changes in renal function (24).

One proposed mechanism underlying the benefits of exercise in CKD is the reduction of chronic systemic inflammation, including modulation of immune cell activity, decreased cytokine release, and upregulation of the NRF2 antioxidant pathway (31). Additionally, cardiovascular improvements play a central role. A recent meta-analysis demonstrated that exercise significantly improves arterial stiffness, as measured by pulse wave velocity and augmentation index, in both patients with pre-dialysis and dialysis CKD (32). This is clinically relevant, as increased arterial stiffness is associated with cardiovascular mortality and faster renal decline in CKD (33,34). Mechanistically, exercise promotes laminar shear stress, enhancing nitric oxide production and reducing oxidative stress (35). These data have informed the UK Renal Association's guidelines on physical activity and lifestyle for patients with CKD at all stages (3).

In the present study, physically active patients reported higher scores in seven of the eight SF-36 QoL domains, with statistically significant differences observed in functional capacity, physical functioning, pain, general health, vitality, and mental health. These results are consistent with those in the literature demonstrating that regular physical activity is associated with better QoL perceptions in patients with CKD (9). Active patients also performed better in objective functional capacity tests, reinforcing that physical activity enhances not only perceived well-being but also measurable physical functions (9,36).

Statistically significant correlations between PAL and five SF-36 domains were identified, particularly vitality and general health, indicating that physical activity substantially contributes to improved QoL in stages 3b and 4 of CKD. These findings are supported by a recent systematic review linking low physical function and inactivity to higher mortality and adverse outcomes in patients with non-dialysis CKD, although causality remains to be established (37). Similarly, in Italian hemodialysis patients, physical function was the most powerful predictor of mortality among the SF-36 domains (38).

The novelty of our study lies in the comprehensive assessment of physical activity, combining both self-reported measures and objective physical tests in patients with pre-dialysis CKD, a population in which the implementation of targeted intervention strategies may help delay progression to hemodialysis. Additionally, we examined the impact of PALs on QoL and the risk of CKD progression. Another important strength of our study is the use of questionnaires that have been previously validated for the Brazilian population, particularly the IPAQ, which is appropriate for the age group represented in our sample. As noted in the literature, most studies involving patients with pre-dialysis CKD have assessed physical activity exclusively through questionnaires, typically the IPAQ (11,12,39), or have not evaluated the impact of physical activity on QoL (6).

Tsai et al. (9) conducted an interesting study that included patients across all CKD stages and assessed physical activity using both questionnaires and objective physical tests (handgrip strength, 30-s chair stand, and 2-min step), correlating these measures with QoL parameters. However, their approach differed from ours in that patients were grouped into broader categories (stages 1-3 and 4-5), and different instruments were used to assess physical activity and QoL.

Clinically, our findings emphasize the importance of assessing and addressing physical activity and QoL in patients with CKD stages 3b and 4. Interventions may include tailored exercise prescriptions, professional supervision, family and community support, health education, and motivational strategies to foster long-term adherence to active lifestyles.

This study has some limitations. The sample size and recruitment from a single outpatient center may limit the generalizability of the study. The use of convenience sampling may introduce selection bias. Additionally, PAL was assessed using a self-reported questionnaire, which is subject to recall bias and may overestimate or underestimate true activity levels. Finally, the cross-sectional design precludes causal inference. Future longitudinal studies with larger and more diverse samples are necessary to elucidate the causal relationships among physical activity, QoL, and CKD progression.

In conclusion, physical inactivity is prevalent among patients with CKD stages 3b and 4, and physically active individuals report better QoL and exhibit lower levels of albuminuria, which are key indicators in mitigating CKD progression. These findings underscore the importance of integrating physical activity promotion into the management of patients with pre-dialysis CKD. Multidisciplinary teams should explore strategies to encourage and sustain active lifestyles, including collaboration with physical education and nutrition professionals. Further studies are needed to determine the most effective exercise modalities for improving outcomes in patients with non-dialysis advanced CKD.

## Data Availability

All data generated or analyzed during this study can be made available upon reasonable request.
